# *SLC12A3* variants modulate LDL cholesterol levels in the Mongolian population

**DOI:** 10.1186/s12944-017-0413-x

**Published:** 2017-02-06

**Authors:** Caiyan An, Kejin Zhang, Xiulan Su

**Affiliations:** 1 Clinical Research Center of the Affiliated Hospital, Inner Mongolia Medical University, Hohhot, 010050 Inner Mongolia China; 2 Key Laboratory of Resource Biology and Biotechnology in Western China (Ministry of Education), College of Life Science, Institute of Population and Health, Northwest University, Xi’an, China

**Keywords:** Lipid metabolism, Low-density lipoprotein cholesterol (LDL-c), *SLC12A3*, Mongolians, Haplotype

## Abstract

**Background:**

Abnormalities in lipid metabolism are crucial factors in the pathogenesis of cardiovascular disease (CVD). Variants of many genes have been verified to confer risk for lipid metabolism abnormalities. However, the relationship between genetic variants of the NCC-encoding *SLC12A3* gene and lipid metabolism in the Mongolian population remains unclear. In the present study, we aimed to elucidate the effects of *SLC12A3* variants on Mongolian lipid metabolism, including total cholesterol (TCHO), triglycerides (TG), low-density lipoprotein cholesterol (LDL-c), and high-density lipoprotein cholesterol (HDL-c).

**Methods:**

A randomly selected population of Mongolians (n = 331) from China underwent clinical testing. An ANOVA test, Kruskal-Wallis *H* test (K-W test) and haplotype analysis were used to evaluate the association between the levels of lipids (TCHO, TG, LDL-c, and HDL-c) and polymorphisms in *SLC12A3* loci.

**Results:**

We identified three single nucleotide polymorphisms (SNPs) rs5803, rs2010501 and rs711746 in the *SLC12A3* gene that were significantly associated with an individual’s serum LDL-c level. Haplotypes combining these SNPs also showed the same trend (all *p* values < 0.01). Furthermore, the influence of *SLC12A3* genetic polymorphisms on differences in individual serum LDL-c levels remained significant, even after we controlled gender, and demographic and other non-genetic factors.

**Conclusion:**

These results suggest that variants of the *SLC12A3* gene confer susceptibility to the abnormal serum LDL-c level in the Mongolian population.

## Background

Serum lipid and lipoprotein concentrations are tightly associated with cardiovascular disease (CVD), which is the major leading cause of death and disability worldwide [1]. Abnormalities in lipid metabolism, such as increased levels of triglycerides (TG) and low-density lipoprotein cholesterol(LDL-c), and decreased levels of high-density lipoprotein cholesterol(HDL-c), have been identified to be crucial factors to the pathogenesis of CVD [2, 3]. Many well-designed studies have clearly established LDL-c as the major target for lipid-modifying therapy [4] because elevated LDL-c is a major contributor to CVD [5, 6].

Serum lipid levels are also modulated by genetic [7, 8] and multiple environmental risk factors [9, 10] and their interactions [11, 12]. Approximately 50% of serum lipid abnormalities can be explained by genetic variants [13]. For instance, Chen et al. [14] proposed that there was a close relationship between the *MTHFR* gene and the longevity of a cohort in Bama, a well-known home of longevity in China, and that T allele carriers had a modestly unfavorable impact on lipid levels (i.e., higher LDL-c level) with a gender difference. Sone et al. [15] discovered that the genetic variants of the *FADS* gene are associated with LDL-c level in Japanese males, and Cuevas et al. [16] suggested the *HMGCR* rs17671591 polymorphism as a genetic marker of lower LDL-c after atorvastatin therapy in the Chilean population.

In addition to genetic predisposition, epidemiological risk factors also play crucial roles in the abnormalities of lipid profiles, including, 1) gender differences, a common feature in an individual’s serum lipid levels, and 2) body mass index (BMI, weight in kilograms divided by height in square meters). Garcia-Palmieri et al. [17] stated that diet and relative weight could account for up to 6% of the variability in serum cholesterol levels. To be more specific, for every 1-kg decrease in body weight, TG decreased by 0.011 mmol/L and HDL-c increased by 0.011 mmol/L [18]; 3) Lifestyle (e.g., smoking, alcohol consumption, etc.) has also been shown to influence serum lipid levels. Rimm et al. [19] documented that consuming 30 g of ethanol per day increased the concentrations of HDL-c by 3.99 mg/dL, and TG by 5.69 mg/dL; Yin et al. [11, 20] also showed that BMI, cigarette smoking and alcohol consumption could interact with certain lipid-related gene variants to modify the serum lipid levels in BaiKu Yao and Han Chinese ethnic groups.

Mongolian is one of the ethnic groups in China with a high prevalence of hypertension and lipid abnormalities. Family aggregation of the diseases in Mongolians suggests that the genetic factor might play an important role in the etiology of these diseases in Mongolians. The genetic basis of lipid abnormality is complex; therefore, it is a huge challenge for us to certify those true susceptible genes/loci to lipid abnormality. Carrying out association studies in some population with different evolutionary history and linkage disequilbrium (LD), such as minor ethnic groups, may further narrow these regions to identify the causal gene(s) and is helpful for the identification of functional variants of complex diseases.

Our previous studies demonstrated the association of *SLC12A3* gene variants with Mongolian hypertension [21–23] and meanwhile we also found that the levels of TC [21], TG [21–23] and LDL-c [21, 23] are significantly increased, whereas that of HDL-c [21] is slightly decreased in Mongolian hypertension when compared with Mongolian normotensives. Thus, it is possible that the genetic polymorphisms of *SLC12A3* might be associated with hypertension via modulating an individual’s serum lipid levels. This possibility was tested on a randomly selected population of Mongolians (n = 331) from China and at the present study we aimed to clarify the association of *SLC12A3* variants with lipid profiles and the mechanisms underlying lipid abnormality in Mongolians and provide the scientific basis for the prevention and treatment of this complex disease.

## Methods

### Subjects

We tested 331 unrelated, randomly selected adult residents (59.81% female, mean age = 45 ± 12.17 years (range 20–70 years old)) in the Inner Mongolia Autonomous Region of China, including Dongwuzhumuqin County, Xianghuang County, and the city of Xilinhot. All subjects were of Mongolian ethnic origins. Written informed consents were obtained from all participants. The study was performed in accordance with the Declaration of Helsinki and was approved by the Ethical Committee of Affiliated Hospital of Inner Mongolia Medical University.

### Demographic information and serum lipid parameters

Demographic variables collected in this study included age, gender, smoking status and drinking status. A smoker was defined as smoking at least one cigarette per day for at least 1 year, and smoking status was categorized into never (76.44%) and current (20.24%) subgroups. Alcohol consumption was defined as current status (20.54%), consuming 50 mL or more alcohol per day for at least 1 year. Participants’ heart rate, or pulse, was measured as the number heart beats per minute. Subjects’ height and weight were measured, body mass index (BMI) was calculated as weight (kg) divided by the square of height (m), and waist-hip ratio (WHR) was assessed by dividing waistline (cm) by hipline (cm). Normal weight, overweight, moderate obesity, and severe obesity were defined by BMI and WHR indexes according to WHO recommendations.

Blood samples were obtained from the antecubital vein after ≥8 h of fasting. Part of the collected samples were used to determine serum lipid levels and another part were transferred into a tube with anti-coagulate solution and used to extract deoxyribonucleic acid (DNA). To profile individuals’ serum lipid status, four important parameters, TCHO, TG, HDL-c, and LDL-c were measured within 8 h, using routine methods in the local hospital. Fasting blood glucose was tested using a Dollar general glucose meter (Dollar, Korea). Genomic DNA was isolated from peripheral blood leukocytes using an AxyPrep-96 DNA Extraction Kit (Axygen, Union City, CA, USA).

### Tag SNPs and genotyping

To minimize the genotyping load while maximizing association information, the implementation of haplotype-tagging SNP (tSNP) selection was performed using the HAPLOVIEW 4.2 software package, using the method described by Gabriel et al.[24]. Fifteen SNPs of the *SLC12A3* gene were chosen from HCB_Asian population data in the HapMap SNP set (version 22) (http://hapmap.ncbi.nlm.nih.gov/) based on pairwise *r* ^2^ ≥ 0.5 and minor allele frequency (MAF) ≥ 0.05.

The genotyping was performed as described previously [22]. In brief, 1) the target DNA sequences were amplified using a multiplex PCR method in a final volume of 10 μl; 2) thermal cycling was performed for all SNPs loci in Gene Amp PCR system 9600 (PerkinElmer, Waltham, MA, USA); and 3) the fluorescent products of ligase detection reaction were differentiated by Applied Biosystems 3730 DNA Analyzer (Applied Biosystems, CA, USA). To verify the accuracy of the genotyping results for tagSNPs, we randomly sequenced 20 samples.

### Statistical analysis

PASW statistics 18 (formerly SPSS Statistics; http://www.spss.com.hk/statistics) was used to analyze the data. The Hardy-Weinberg equilibrium (HWE) testing was calculated using the Finetti method (https://ihg.gsf.de/cgi-bin/hw/hwa2.pl), and the gender differences for alleles and genotypes’ distributions were also evaluated. All variables were categorized by the median value in study sample and *t*-tests were performed to estimate the effects of non-genetic variables on individuals’ serum lipid levels. A one-way analysis of variance (ANOVA) and *LSD post hoc* test were performed to examine the influence of each tagSNP on individual serum lipid status (including TCHO, TG, HDL-c and LDL-c). The non-parametric test, Kruskal-Wallis *H* test (K-W test) was also used. Linkage condition and haplotypes construction of positive SNPs were estimated by UNPHASED software (version 3.0.13) [25]. More stringent criteria for positive tag SNP(s) was adopted: tag SNP(s) which demonstrated a significant association using both ANOVA and the K-W test were considered.

To partial out other non-genetic variables (i.e., age, gender, BMI, and WHR) that might have influences on the relationship between SNPs and individuals’ serum lipid status, a multiple regression analysis was performed in which the genotype group served as one of the class variables. For the multiple linear regression analysis, we performed the following steps: (step 1) entering control variable(s) and (step 2) entering both variables and the genetic information. The statistical power analysis was referred to as *p* < 0.05 for two-tailed tests.

## Results

### Demographic characteristics and serum lipid levels

The general characteristics of subjects and their relationship with individuals’ serum lipid levels were presented in Table 1. Comparison tests found that serum lipid profiles were correlated with several environmental factors, including age, gender, alcohol consumption, cigarette smoking, blood glucose, BMI and WHR (*p* < 0.05–0.001). Older subjects (>45 years) showed a significantly higher serum level of TG than that of younger subjects (≤ 45 years) (1.98 ± 2.42 vs.1.51 ± 1.26, *p* < 0.05). Men had higher serum levels of TCHO, TG and LDL-c than women (all *p*s < 0.01–0.001). Smokers also had higher serum levels of LDL-c than never smokers (3.52 ± 1.37, *p* < 0.01). Alcohol consumption also exhibited significantly higher serum levels of TCHO, TG and LDL-c than never drinkers (all *p* values < 0.05–0.01). The subjects with higher glucose levels in blood had extremely higher serum levels of TCHO, HDL-c, LDL-c (all *p* values < 0.001), and higher levels of TG (*p* < 0.05) than those with normal glucose. Lower heart rate per minute also showed a weak influence on individuals’ TCHO and HDL-c levels (all *p* values < 0.05). Over-weight and obesity (BMI > 25.2 kg/m^2^, or WHR > 0.88) often yielded significantly higher levels of TG and HDL-c (all *p* values < 0.05–0.001), whereas normal BMI was associated with a significantly high HDL-c level (*p* < 0.001).

**Table 1:** Effects of demographic, lifestyle characteristics and other non-genetic factors on individuals’ serum lipid levels

Variables ^a^	n	TCHO (mmol/L)	TG (mmol/L)	HDL-c (mmol/L)	LDL-c (mmol/L)
Age (years)					
≤ 45	171	4.76 ± 1.77	1.51 ± 1.26	1.49 ± 0.58	3.01 ± 1.32
> 45	160	5.08 ± 2.06	1.98 ± 2.42*	1.43 ± 0.55	3.25 ± 1.33
Sex					
Male	139	5.17 ± 1.89***	2.00 ± 2.00**	1.45 ± 0.58	3.34 ± 1.34**
Female	192	4.67 ± 1.71	1.58 ± 1.36	1.45 ± 0.47	2.98 ± 1.28
Smoker					
Never	253	4.85 ± 1.92	1.68 ± 2.03	1.47 ± 0.54	3.05 ± 1.30
Smoker	67	5.31 ± 1.96	1.95 ± 1.49	1.47 ± 0.66	3.52 ± 1.37**
Unknown	11	-	-	-	-
Drink					
Never	241	4.75 ± 1.76	1.59 ± 1.43	1.44 ± 0.53	3.03 ± 1.30
Drinker	68	5.63 ± 2.44**	2.39 ± 3.17**	1.55 ± 0.74	3.54 ± 1.44*
Unknown	22	-	-	-	-
Blood_G (mg/ml)					
≤5.7	160	4.38 ± 1.71	1.50 ± 2.10	1.32 ± 0.47	2.79 ± 1.16
>5.7	147	5.47 ± 2.00***	1.99 ± 1.67*	1.61 ± 0.65***	3.44 ± 1.39***
Unknown	24	-	-	-	-
Heart_R (min^−1^)					
≤ 76	143	5.28 ± 2.11*	1.85 ± 2.25	1.58 ± 0.65*	3.30 ± 1.37
> 76	122	4.75 ± 1.89	1.72 ± 1.65	1.41 ± 0.54	3.07 ± 1.32
Unknown	66	-	-	-	-
BMI (kg/m^2^)					
≤ 24.44	166	4.84 ± 1.95	1.40 ± 1.20	1.60 ± 0.63***	2.91 ± 1.35
> 24.44	162	4.97 ± 1.91	2.07 ± 2.39**	1.32 ± 0.47	3.33 ± 1.27**
Unknown	3	-	-	-	-
WHR					
≤ 0.87	132	4.82 ± 2.07	1.40 ± 1.20	1.54 ± 0.68	3.00 ± 1.35
> 0.87	128	5.23 ± 2.00	2.27 ± 2.53***	1.42 ± 0.54	3.36 ± 1.36*
Unknown	71	-	-	-	-

*** *p* value < 0.001; ** *p* value < 0.01; * *p* value < 0.05

*Abbreviation BMI* body mass index, *WHR* waist-hip ratio, *Blood_G* fasting blood glucose in serum, *Heart_R* heart rate per minute, *TCHO* total cholesterol (mmol/L), *TG* triglycerides (mmol/L), *HDL-C* high-density lipoprotein cholesterol (mmol/L), *LDL-C* low-density lipoprotein cholesterol (mmol/L)

^a^all variables were categorized by the median value in study sample

### Genotyping and distribution analysis

Genotype frequencies of all 15 SNPs showed no significant deviations from Hardy – Weinberg equilibrium (WHE), except rs2289119 and rs7204044 (*X* ^2^ = 3.92, *p* = 0.048, and *X* ^2^ = 7.27, *p* = 0.007, respectively). And no gender difference distributions of alleles and genotypes frequency were found. The genotyping success rates for all SNPs were higher than 96.98% (321/331).

### Genetic variants effects on TCHO, TG, HDL-c and LDL-c

Table 2 presented data of the genetic variants of the *SLC12A3* gene on individuals’ serum lipid profile. Rs5803 polymorphisms showed a significant association with individuals’ serum level of LDL-c (*F* = 3.881, *p* = 0.022 for ANOVA, and *X* ^2^ = 7.378, *p* = 0.025 for K-W test), the T allele carriers had higher LDL-c levels than CC genotype carriers (3.32 ± 1.38 mmol/L vs.2.93 ± 1.25 mmol/L). Both rs2010501 and rs711746 had a close association with individuals’ serum TCHO and LDL-c levels (all *p*s < 0.01). Interestingly, when dominant genetic models (carriers of rare allele vs. dominant allele homozygous) were performed for all SNPs, the results demonstrated the same trend.

**Table 2:** Effects of SNPs on individuals’ serum lipid levels

SNPs ^a^	Variables	Genotypes/Mean (SD)			ANOVA		K-W	
					*F*	*p* ^b^	*Chi* ^2^	*p* ^b^
rs5803		TT(25)	TC(126)	CC(177)				
	TCHO	4.67 ± 1.80	5.21 ± 1.93	4.72 ± 1.93	2.591	0.076	**6.536**	**0.038**
	TG	1.59 ± 1.41	1.77 ± 1.48	1.72 ± 2.25	0.098	0.907	4.247	0.120
	HDL-c	1.49 ± 0.60	1.53 ± 0.57	1.42 ± 0.55	1.470	0.231	4.256	0.119
	LDL-c	3.10 ± 1.41	3.36 ± 1.38	2.93 ± 1.25	**3.881**	**0.022**	**7.378**	**0.025**
rs2010501		CC(16)	CT(110)	TT(195)				
	TCHO	4.07 ± 1.28	4.70 ± 1.97	5.12 ± 1.94	**3.390**	**0.035**	**7.014**	**0.030**
	TG	1.07 ± 0.66	1.64 ± 2.51	1.86 ± 1.62	1.492	0.227	**13.604**	**0.001**
	HDL-c	1.35 ± 0.54	1.45 ± 0.59	1.50 ± 0.56	0.620	0.538	1.690	0.430
	LDL-c	2.49 ± 0.86	2.87 ± 1.28	3.31 ± 1.37	**5.868**	**0.003**	**11.173**	**0.004**
rs711746		GG(48)	GA(148)	AA(125)				
	TCHO	4.14 ± 1.17	5.00 ± 2.01	5.14 ± 2.04	**4.910**	**0.008**	**7.553**	**0.023**
	TG	1.18 ± 0.48	1.83 ± 2.38	1.87 ± 1.61	2.444	0.088	**11.284**	**0.004**
	HDL-c	1.37 ± 0.48	1.49 ± 0.59	1.50 ± 0.57	1.020	0.362	2.177	0.337
	LDL-c	2.55 ± 0.85	3.10 ± 1.35	3.35 ± 1.42	**6.333**	**0.002**	**9.911**	**0.007**

*Abbreviation*: *TCHO* total cholesterol (mmol/L), *TG* triglyceride (mmol/L), *HDL-c* high-density lipoprotein cholesterol (mmol/L), *LDL-c* low-density lipoprotein cholesterol (mmol/L), *ANOVA* one-way analysis of variance, *K-W* Kruskal-Wallis *H* test

^a^SNPs, at least one positive association between individuals’ serum levels presented, were shown analyzed by both ANOVA and K-W tests

^b^Bold type denotes *p* < 0.05

To confirm the above analyses, we also derived the predicted probabilities of being single for different genotypes from the linear regression equation, after controlling other variables. Finally, the association between rs5803, rs2010501, rs711746 and individuals’ serum level of LDL-c was found to still be significant, even with other variables controlled (*B* = -0.044, *Error SD* = 0.018, *t* = -2.490, *p* = 0.014 for *rs5803, B* = 0.048, *Error SD* = 0.018, *t* = 2.653, *p* = 0.009 for rs2010501, and *B* = 0.040, *Error SD* = 0.017, *t* = 2.350, *p* = 0.020 for rs711746). The T allele of rs5803 had a negative association with individuals’ elevated serum level of LDL-c, whereas the T allele of rs2010501 and A allele of rs711746 with a positive association. The genetic polymorphisms of three SNPs contributed more than 11.9% variation of individual’s serum LDL-c level difference (*R* ^2^ change > 0.119, and *Sig.* of *F* change < 0.001, for both SNPs). Additionally, individuals’ levels of blood glucose were found to have an extremely significant effect (all *p*s < 0.001).

### Haplotype analysis for positive SNPs

The linkage disequilibrium among the three positive SNPs was estimated by UNPHASED software, and a strong linkage disequilibrium between rs2010501 and rs711746 (*D*’ = 0.97, *r* ^2^ = 0.44) was observed. We constructed three sets of haplotypes. Two were derived from various combinations of two SNPs (two window size), and one was derived from a combination of all three SNPs (three window size). Three sets of haplotypes displayed significant additive genetic influences on individual’s serum LDL-c levels in both two and three windows analyses (all global *p* values < 0.001). Several individual haplotypes, constructed by two or three SNP sites, demonstrated significant genetic effects on individuals’ serum LDL-c levels, compared with the first haplotypes (all *p* values < 0.05–0.001) (Table 3).

**Table 3:** Effects of haplotypes constructed by three positive SNPs on LDL-c levels

Haplotypes ^a^	Frequency (%)	AddVal	95%Lo	95%Hi	Chisq	*p* ^b^	Global *p* ^b^
	rs5803(T/C)-rs2010501(C/T)						
T-T	22.43	−0.044	−0.042	0.335	4.927	**0.026**	**2.23e-4**
C-C	18.65	−0.448	−0.852	−0.043	14.03	**1.80e-4**	
C-T	55.25	−0.105	−0.453	0.243	1.002	0.317	
	rs2010501(C/T)-rs711746(G/A)						
C-G	21.81	-	-	-	10.56	**1.15e-3**	**7.68e-4**
T-G	16.16	0.145	−0.065	0.355	0.734	0.391	
T-A	61.65	0.291	0.128	0.453	12.56	**3.95e-4**	
	rs5803(T/C)-rs2010501(C/T)-rs711746(G/A)						
T-T-A	21.90	−0.050	−0.502	0.402	4.521	**0.033**	**5.76e-4**
C-C-G	18.61	−0.426	−0.898	0.045	12.43	**4.22e-4**	
C-T-G	15.40	−0.224	−0.627	0.180	0.967	0.325	
C-T-A	39.52	−0.062	−0.509	0.386	3.035	0.812	

*Abbreviation*: *Fre*. is the frequencies of risk haplotypes, *AddVal* the estimated additive genetic value relative to the reference haplotype, *95% Lo* 95% lower confidence limit for the additive value, *95% Hi* 95% upper confidence limit for the additive value

^a^The haplotypes frequencies < 0.05 were excluded in global *p*-value calculation

^b^Bold type denotes *p* < 0.05

## Discussion

In this study, we investigated the influence of genetic polymorphisms in the *SLC12A3* gene on human serum lipid levels (TCHO, TG, HDL-c and LDL-c), within a randomly selected Mongolian cohort in China. Our data demonstrated that three SNPs, rs5803, rs2010501 and rs711746 of 15 tag SNPs in the *SLC12A3* gene were significantly associated with individuals’ serum levels of LDL-c. Haplotypes combining with these SNPs also showed the same trend. Furthermore, the influence of *SLC12A3* genetic polymorphisms on the differences observed in individual serum LDL-c levels remained significant, even after we controlled demographic, gender and other non-genetic factors. To the best of our knowledge, this study is the first to demonstrate the influence of genetic variants in *SLC12A3* on human serum level of LDL-c.

LDL-c plays a pivotal role in CVD, while the association between elevated serum LDL-c level and hypertension remains unclear [26–28]. We observed an elevated serum LDL-c level in hypertensive patients [21, 23] when we investigated the association between genetic variants of *SLC12A3* and hypertensive patients in Mongolians in China. The results from this randomly selected Mongolian sample were consistent with our hypothesis that *SLC12A3* polymorphisms may modulate serum LDL-c level and represent a close relationship with hypertensive patients that have been reported previously [22, 23]. Two SNPs (rs5803 and rs711746), which were reportedly associated with hypertension in case-control and family-based association studies, also showed significant influences on individuals’ serum LDL-c level in the general Mongolian population. Collectively, these results demonstrated the important modulation effect of *SLC13A* gene on human serum LDL-c levels and their observed association with hypertension.

Demographic and lifestyle characteristics also showed a close relation with serum LDL-c levels, in addition to its genetic association. In the present population, several non-genetic variables, including gender, smoking status, alcohol consumption, fasting blood glucose, BMI and WHR, all contributed to differences in individuals’ serum levels of LDL-c (Table 2). Meanwhile, gender difference may play an important role and should be considered further in future experiments. Lifestyle factors, including smoking status and alcohol consumption revealed an extreme difference between males and females. Males also demonstrated higher BMI and WHR indexes than females (data not shown). However, when we used linear regression analysis to estimate each variable’s influence on individuals’ LDL-c level, the impact of blood glucose concentration on LDL-c was observed, whereas the influence from other variables disappeared, except for the three genetic variants of *SLC12A3*. Hsu and Fava et al. [29, 30] also suspected that *SLC12A3* polymorphisms might regulate individuals’ blood pressure via affecting their fasting plasma glucose concentrations.

One should be cautious when interpreting these findings given the following limitations. We examined the influence of genetic polymorphisms in the *SLC12A3* gene on human LDL-c level status within a specific population. The high prevalence of hypertension is a serious health problem among Mongolian people [31], as CVD is a primary cause of morbidity and mortality [32]. Genetic influences of the *SLC12A3* gene on LDL-c levels were revealed in this population. Second, the limited sample size should also be carefully noted, despite the statistical power analyses showing a satisfied estimation value. For some rare homozygous alleles, the sample size still seemed insufficient, especially when we partialed out subjects with missing variables. Third, several characteristics and lifestyle information were included in this study. These characteristics are susceptible to change, and it is not clear whether the association between the *SLC12A3* and LDL-c level would still hold. It may be the case that under certain circumstances, the genetic contribution to LDL-c level could be overshadowed by other factors. In addition, to avoid false association a double-measure was performed in our study, that is, the association between polymorphisms of *SLC12A3* and lipid metabolism will be considered, only when both the parametric (ANOVA) and non-parametric (KW) analyses results were supportive and consistent. However, we couldn’t rule out the impact of *SLC12A3* on individual’s TCHO and TG levels, although it did not show a consistent correlation, completely.

## Conclusion

Our results concluded that variants of the *SLC12A3* gene confer susceptibility to the abnormal serum LDL-c levels in the Mongolian population.

## Abbreviations

ANOVA: One-way analysis of variance
BMI: Body mass index
CVD: Cardiovascular disease
HDL-C: High-density lipoprotein cholesterol
K-W: Kruskal-Wallis H test
LDL-C: Low-density lipoprotein cholesterol
MAF: Minor allele frequency
TCHO: Total cholesterol
TG: Triglycerides
WHR: waist-hip ratio
